# Prevalence and Characterization of *Staphylococcus aureus* Cultured From Raw Milk Taken From Dairy Cows With Mastitis in Beijing, China

**DOI:** 10.3389/fmicb.2018.01123

**Published:** 2018-06-22

**Authors:** Wei Wang, Xiaohui Lin, Tao Jiang, Zixin Peng, Jin Xu, Lingxian Yi, Fengqin Li, Séamus Fanning, Zulqarnain Baloch

**Affiliations:** ^1^Key Laboratory of Food Safety Risk Assessment, Ministry of Health, China National Center for Food Safety Risk Assessment, Beijing, China; ^2^Physics and Chemical Department, Tianjin Center for Disease Control and Prevention, Tianjin, China; ^3^College of Veterinary Medicine, South China Agricultural University, Guangzhou, China; ^4^UCD-Centre for Food Safety, School of Public Health, Physiotherapy and Sports Science, University College Dublin, Dublin, Ireland; ^5^School of Biological Sciences, Institute for Global Food Security, Queen's University Belfast, Belfast, United Kingdom

**Keywords:** *Staphylococcus aureus*, raw milk, mastitis, antimicrobial susceptible test, virulence factors, enterotoxin production, biofilm, molecular typing

## Abstract

The colonization of dairy herds and subsequent contamination of raw milk by *Staphylococcus aureus* (*S. aureus*), especially those expressing a multi-drug resistance (MDR), biofilm and toxins producing ability, remains an important issue for both the dairy producer and public health. In this study, we investigated the prevalence, antimicrobial resistance, virulence, and genetic diversity of *S. aureus* in raw milk taken from 2 dairy farms in Beijing, China. Ninety (46.2%, 90/195) samples were positive for *S. aureus*. Resistant to penicillin (PEN) (31.3%), ciprofloxacin (18.8%) and enrofloxacin (15.6%) were the most often observed. Isolates cultured from farm B showed significantly higher resistance to penicillin (73.9%), ciprofloxacin (34.8%), enrofloxacin (34.8%), tilmicosin (17.4%), and erythromycin (17.4%) than those from farm A (*p* < 0.05). Totally, 94.8% *S. aureus* harbored at least one virulence gene and the *pvl* (93.8%), *sec* (65.6%), and *sea* (60.4%) genes were the most frequently detected. The *pvl* and *sec* genes were more often detected in isolates from farm A (97.3% and 84.9% respectively) than those from farm B (*p* < 0.05). Of all 77 staphylococcus enterotoxin (SE)-positive isolates, more than 90% could produce enterotoxins and 70.1% could produce two types. Biofilm related genes (*icaA*/*D, clf/B, can*, and *fnbA*) were detected in all96 isolates. All 96 isolates could produce biofilm with 8.3, 70.8, and 18.8% of the isolates demonstrating weak, moderate and strong biofilm formation, respectively. A total of 5 STs, 7 *spa* types (1 novel *spa* type t17182), 3*agr* types (no *agr*II), and 14 *SmaI*-pulso-types were found in this study. PFGE cluster II-CC1-ST1-t127-*agr* III was the most prevalent clone (56.3%). Isolates of *agr* III (PFGE Cluster I/II-CC1-ST1-t127/2279) had higher detection of virulence genes than those of *agr* I and *agr* IV. TheMSSA-ST398-t1456-*agr* I clone expressed the greatest MDRbut with no virulence genes and weakly biofilm formation. Our finding indicated a relatively high prevalence of *S. aureus* with less antimicrobial resistance but often positive for enterotoxigenicity and biofilm formation. This study could help identify predominant clones and provide surveillance measures to eliminate and decrease the contamination of *S. aureus* in raw milk of dairy cows with mastitis.

## Introduction

*Staphylococcus aureus* (*S. aureus*) is one of the leading sources of intra-mammary infections in dairy cows (Dufour et al., 2012; Zecconi and Scali, 2013). It is reported that 10–40% of the mastitis cases are caused by *S. aureus* in China and other countries (Kateete et al., 2013; Basanisi et al., 2017; Liu et al., 2017). Mastitis is a global challenge that it can result in financial losses for the dairy industry and the economy due to the substandard quality of milk, treatment costs, and causing subsequent new infection of other cows (Schroeder, 2012). Contaminated raw milk at farm level, may lead to subsequent problems further along the food chain giving rise to *S. Aureus* associated food contamination (Jakobsen et al., 2011; Rola et al., 2016).

*S. aureus* associated food poisoning in humans and similarly mastitis in animal is caused by those isolates possessing virulence factors (Hennekinne et al., 2012). This bacterium produces wide range of factors, for example toxic shock syndrome toxin-1 (TSST-1), staphylococcus enterotoxin (SE), and Panton-Valentine leukocidin (PVL). SEs is regarded as the major cause of *S. aureus* associated food poisoning (Bergdoll et al., 1981; Hennekinne et al., 2012). It is reported that more than 90% of *S. aureus*-associated food poisoning outbreaks were attributed to the classical SEs (denoted as SEA to SEE) encoded by *sea* to *see* genes (Tarekgne et al., 2016). The TSST-1 toxin could result in toxic shock syndrome by reducing the host immune response, while PVL could destruct host leukocyte and cause tissue necrosis (Schlievert et al., 1981).

Antimicrobial therapy is an important strategy for mastitis control as well as human infections (Gomes and Henriques, 2016). However, *S. aureus* often exhibit resistance to multiple classes of antimicrobial agents as a response to the selective pressure of antimicrobials, which will narrow the treatment options for clinicians and veterinarians (Gomes and Henriques, 2016). It is reported that many *S. aureus*-associated food poisoning outbreaks were due to multi-drug resistant (MDR) *S. aureus* including methicillin-resistant *S. aureus* (MRSA) (Johler et al., 2015; Jans et al., 2017). Furthermore, formation of biofilms, highly organized multicellular complexes, is often associated with both epithelial adhesion and evasion of host immune defenses (Melchior et al., 2009). Biofilm associated protein (Bap) plays an important role in primary attachment and recruitment of *S. aureus* (Khoramian et al., 2015; Felipe et al., 2017). The *icaA* and *icaD* genes that form part of the *icaABCD* gene cluster (intracellular adhesion locus) are essential for biofilm formation (Khoramian et al., 2015; Felipe et al., 2017). Additionally, the collagen binding proteins (Cna), clumping factors (ClfA and ClfB) and fibronectin binding proteins (FnbA and FnbB) also have associations with biofilm production according to previous studies (Khoramian et al., 2015; Pereyra et al., 2016).

Molecular epidemiology-based methods are essential tools for the study of clonal relatedness, genetic diversity, and also tracking the dissemination of *S. aureus* infections. It was reported that certain *S. aureus* lineages were specifically associated with milk, such as CC97 (Clonal complex), and particular clonal lineages may be prevalent geographically, and have specific antimicrobial resistance and virulence patterns (Hata et al., 2010). This study aimed to estimate the prevalence of *S. aureus* among raw milk from dairy cows with clinical mastitis from two dairy farms during August to December in 2016 in Beijing, China, and to describe the characteristics of the isolates, in order to provide groundwork for further studies on the control and prevention of contamination of *S. aureus* in raw milk of dairy cows with mastitis.

## Materials and methods

### Sampling and isolation of *S. aureus*

Recruitment of cows into this study was done in consultation with veterinarians and sampling process was carried on with the agreement of the dairy farms' owners. Raw milk samples were collected from cows presenting with clinical mastitis consistent with poor milk yield, color change and udders inflammation. Milk collection process was performed after cleaning the teats, initial streams of milk discarded and teat tips scrubbed with cotton balls moistened with 75% alcohol. Teat-cleaning before milking and treatment with antibiotics at dry-off were not performed. In total, one milk sample from each cow was collected and 195 individual milk samples of 195 cows were obtained from 2 dairy farms during August to December in 2016 in Beijing, China. These two dairy farms belong to one of the largest dairy production companies in China, which mainly supply consumers in Beijing and other regions in China, and also export internationally. Both farms were managed with an intensive breeding system, with the herd size of about 500 locating cows.

The *S. aureus* contamination was detected in raw milk samples according to National Food Safety Standards of China document GB 4789.10-2016. Briefly, a 25-ml milk sample was taken and mixed thoroughly, and then transferred into 225 mL 10% (w/v%) saline solution (Land Bridge, Beijing, China) and homogenize it and solutions were incubated at 37°C for 24 h. A loopful of the incubated culture were streaked onto Baird-Parker Agar supplemented with 5% egg yolk and tellurite, and Blood Agar with sterile defibrinated sheep blood (Land Bridge, Beijing, China), respectively, then incubated at 37°C for 24–48 h. Putative *S. aureus* isolates were tested for coagulase activity, and were further confirmed using API STAPH test strips (bio-Mérieux, Marcyl'Etoile, France). Finally, all isolates were subjected the detection of 16SrRNA and *nuc* genes by PCR (Table 1; Murakami et al., 1991). All confirmed *S. aureus* isolates were stored in BHI with 40% [v/v%] glycerol (Land Bridge, Beijing, China) at −80°C. No more than2 isolates of each sample were chose for subsequent studies.

**Table 1:** Primers used in this study.

**Gene**	**Oligonucleotide sequence (5′−3′)**	**Size of product**	**Annealing temperature**	**References**
*nuc*-F	GCGATTGATGGTGATACGGTT	798	55	Murakami et al., 1991
*nuc*-R	AGCCAAGCCTTGACGAACTAAAGC			
*16S rRNA*-F	AGAGTTTGATCATGGCTCAG	270	55	
*16S rRNA*-R	GGACTACCAGGGTATCTAAT			
*mecA*-F	AAAATCGATGGTAAAGGTTGGC	533	55	
*mecA*-R	AGTTCTGCAGTACCGGATTTGC			
*sea*-F	ACGATCAATTTTTACAGC	544	44.5	Rosec and Gigaud, 2002
*sea*-R	TGCATGTTTTCAGAGTTAATC			
*seb*-F	ATTCTATTAAGGACACTAAGTTAGGGGA	404	44.5	Jarraud et al., 2002
*seb*-R	ATCCCGTTTCATAAGGCGAGT			
*sec*-F	GACATAAAAGCTAGGAATTT	257	46.2	Rosec and Gigaud, 2002
*sec*-R	AAATCGGATTAACATTATCCA			
*sed*-F	CAAATATATTGATATAATGA	330	44.5	Khoramrooz et al., 2016
*sed*-R	AGTAAAAAAGAGTAATGCAA			
*see*-F	CAAAGAAATGCTTTAAGCAATCTTAGGC	482	44.5	Jarraud et al., 2002
*see*-R	CACCTTACCGCCAAAGCTG			
*tst*-F	ACCCCTGTTCCCTTATCATC	326	54	Khoramrooz et al., 2016
*tst*-R	TTTTCAGTATTTGTAACGCC			
*lukS*/*F*-F	ATCATTAGGTAAAATGTCTGGACATGATCCA	433	55	McClure et al., 2006
*lukS*/*F*-R	GCATCAAGTGTATTGGATAGCAAAAGC			
*pan-agr*	ATGCACATGGTGCACATGC	–	55	Shopsin et al., 2003
*agrI*	GTCACAAGTACTATAAGCTGCGAT	440	55	
*agrII*	GTATTACTAATTGAAAAGTGCCATAGC	573	55	
*agrIII*	CTGTTGAAAAAGTCAACTAAAAGCTC	406	55	
*agrIV*	CGATAATGCCGTAATAC CCG	588	55	
*fnbA*-F	GATACAAACCCAGGTGGTGG	191	52	Zmantar et al., 2008
*fnbA*-R	TGTGCTTGACCATGCTCTTC			
*fnbB*-F	ACGCTCAAGGCGACGGCAAAG	197	62	Pereyra et al., 2016
*fnbB*-R	ACCTTCTGCATGACCTTCTGCACCT			
*clfA*-F	CCGGATCCGTAGCTGCAGATGCACC	1000	60	Zmantar et al., 2008
*clfA*-R	GCTCTAGATCACTCATCAGGTTGTTCAGG			
*clfB*-F	TGCAAGTGCAGATTCCGAAAAAAAC	194	62	Klein et al., 2012
*clfB*-R	CCGTCGGTTGAGGTGTTTCATTTG			
*cna*-F	AAAGCGTTGCCTAGTGGAGAC	192	54	Zmantar et al., 2008
*cna*-R	AGTGCCTTCCCAAACCTTTT			
*bap*-F	CCCTATATCGAAGGTGTAGAATTG	971	60	Darwish and Asfour, 2013
*bap*-R	GCTGTTGAAGTTAATACTGTACCTGC			
*icaA*-F	CCTAACTAACGAAAGGTAG	1351	49	
*icaA*-R	AAGATATAGCGATAAGTGC			
*icaD*-F	AAACGTAAGAGAGGTGG	381	49	Pereyra et al., 2016
*icaD*-R	GGCAATATGATCAAGATAC			

### Antimicrobial susceptibility testing (AST)

In this study, broth dilution method was applied to estimate the antimicrobial susceptibility of all tested isolates using the Biofosun® Gram-positive panel (Fosun Diagnostics, Shanghai, China) and interpreted by the Clinical and Laboratory Standards Institute (CLSI) (CLSI, 2015). The antimicrobial agents included Ceftiofur (EFT) (0.25–64 μg/mL), Chloramphenicol (CHL) (0.5–128 μg/mL), Ciprofloxacin (CIP) (0.125–16 μg/mL), Daptomycin (DAP) (0.06–16 μg/mL), Enrofloxacin (ENO) (0.125–32 μg/mL), Erythromycin (ERY) (0.125–16 μg/mL), Fosfomycin (FOS) (0.5–256 μg/mL), Gentamycin (GEN) (0.5–64 μg/mL), Penicillin (PEN) (0.06–32 μg/mL), Tetracycline (TET) (0.25–64 μg/mL), Tilmicosin (TIL) (0.5–64 μg/mL), and Vancomycin (VAN) (0.06–128 μg/mL). *S. aureus* ATCC™29213 was used as the reference strain for the AST.

### Detection of MRSA, virulence and biofilm related genes

Frozen isolates were cultured overnight at 37°C in BHI (Land Bridge, Beijing, China). The genomic DNA was then extracted with TIANamp Bacterial DNA extraction kit (TianGenDNA Kit DP302, Beijing, China), and the quality of DNA was evaluated by a NanoDrop-2000 spectrophotometer (Thermo Fisher Scientific, NH, USA). Sterile deionized water was used to dilute the extracted DNA to 50 mg/L, which was suitable for real-time PCR assays. The genes encoding the methicillin resistance gene (*mecA*), SEs (*sea* to *see*), toxic-shock syndrome toxin (*tst*), Panton-Valentine leukocidin (*lukF*), biofilm related genes (*bap, icaA*, and *icaD*), and adhesion related genes (*fnbA, fnbB, clfA, clfB*, and *can*) were detected by PCR. The primers were supplied by Thermo Fisher Scientific (Waltham, MA, USA; Table 1). Positive and negative controls were included in all PCRs.

### Detection of SEs production

SEs (SEA to SEE)production was detected by immuno-colloidal gold chromatographic test strips (Longrunbio, Beijing, China). In brief, the supernatant of 24 h cultures of *S. aureus* (1 × 10^9^ CFU/mL) positive with SEs genes grown at 37°C in a shake-tube (Xuzhou Yanjia Glass Products, Xuzhou, China) containing 5 mL BHI (Land Bridge, Beijing, China) was separated from cells by centrifugation at 8,000 × g for 20 min. The supernatant was heated at 100°C for 10 min. Then 200 μL of the heated supernatant were tested for the presence of the SEs by the strip test assay. The samples 100 ng/mL of SEA to SEE were used as a positive control and phosphate buffer was used as negative control.

### Biofilm formation

Biofilm production was assessed by a 96-well microtiter plate assay using minimal medium M9 (6 g/l Na_2_ HPO_4_, 3 g/l KH_2_PO_4_, 0.5 g/l NaCl, 1 g/l NH_4_Cl, 2 mM MgSO_4_, 0.1% glucose, and 0.1 mM CaCl_2_; Müsken et al., 2010). After overnight growth in tryptone soy broth medium (TSB; Oxoid Ltd., Basingstoke, UK), 200 μL of cell suspension diluted to 1:100 was transferred into each microtiter plate well, and the later was incubated at 37°C for 72 h. After three brief washes with 200 μL phosphate-buffered saline (PBS) solution and a 20-min fixation step with 200 μL methanol, all plates were stained with 200 μL 0.4% (wt/vol) crystal violet (CV) for 15 min and washed with 200 μL PBS for another 15 min. The formed biofilm was then dissolved with 200 μL 33% (wt/vol) acetic acid for 30 min. The biofilm formation was measured at 570 nm optical density (OD) in a micro-titer plate reader (Tecan, Mannedorf, Switzerland). *Salmonella* Typhimurium ATCC14028, a strong biofilm-forming strain, was selected as the positive control and sterile TSB was used as negative control for the biofilm production assays (Yan et al., 2015). These biofilm assays were performed in triplicate that included biological duplicates. An OD_570nm_ value of 0.6 was applied as the cutoff point to distinguish between biofilm producer from non-biofilm producer [cut-off (ODc) = average OD plus 3 standard deviation (SD) of negative control]. The biofilm formation was classified as strong+++ (OD_570nm_> 1.8), moderate++ (1.8 > OD_570nm_ >1.2), weak+ (1.2 > OD_570nm_ > 0.6), and negative − (OD_570nm_ < 0.6).

### Multilocus sequence typing (MLST)

All *S. aureus* isolates were examined by MLST, based on the sequencing of 7 housekeeping genes described previously (Enright et al., 2000). Alleles and the sequence type (ST) were assigned according to the *S. aureus* MLST database (http://www.mlst.net/). The STs were then clustered in to clonal complexes (CC) by eBURST v.3 software (http://eburst.mlst.net; Feil et al., 2004).

### *spa* typing

The *spa* typing for all *S. aureus* isolates was performed as described previously (Harmsen et al., 2003). The *spa* repeats and types were assigned by the Bio Numerics software v.7.5 (Applied Math, Belgium). If a *spa* repeat did not match any *spa* types, the sequence of this *spa* was then upload to the Ridom *Spa* Server database (http://spa.ridom.de) to assign a new type.

### *agr* genotyping

The *agr* type of all *S. aureus* isolates was determined using the *agr*-group specific primers (*agr* allele types I–V) and *agr* multiplex PCR as described previously (Table 1).

### Pulsed-field gel electrophoresis (PFGE)

The genetic relationships of all *S. aureus* isolates were established by PFGE (Murchan et al., 2003; Ribot et al., 2006). In brief, the tested isolates were cultured and plugs were prepared. Chromosomal DNA was digested with the endonuclease *Sma*I (20 units/μL, New England Biolabs) at 30°C for 3 h. The electrophoresis was performed in 1% agarose SeaKem Gold gel in the CHEF DR III apparatus (Bio-Rad, Hercules, California z) at 14°C for 19 h. Macro restriction patterns were interpreted by Bio Numerics software v.7.5 (Applied Math, Belgium) by the un weighted pair group method with arithmetic averages (UPGMA). *Salmonella* Braenderup H9812 was used as a standard size marker.

### Simpson's index of diversity calculation

The Simpson's index of diversity (diversity index, DI) was used to evaluate the genetic diversity and discriminatory ability of different typing methods. The formula is as follows:

(1) $$\begin{array}{r} DI=1-\frac{1}{\left[N\left(N-1\right)\right]}\overset{s}{\underset{j-1}{\sum }}n_{j}\left(n_{j}-1\right) \end{array}$$

*n*_*j*_ is the number of isolates belonging to the *j*th type, and *N* is the total number of tested isolates.

### Statistical analysis

The Chi-square test was calculated using SPSS 20.0 (SPSS, Chicago, USA), in order to analyze the differences in the prevalent rates, the proportion of isolates resistant to antimicrobial agents, and the distribution of virulence genes, biofilm related genes, enterotoxin production, and biofilm production ability between two farms. Values of *p* < 0.05 were considered statistically significant.

## Results

### Isolation and identification of *S. aureus*

**Figure 1:**
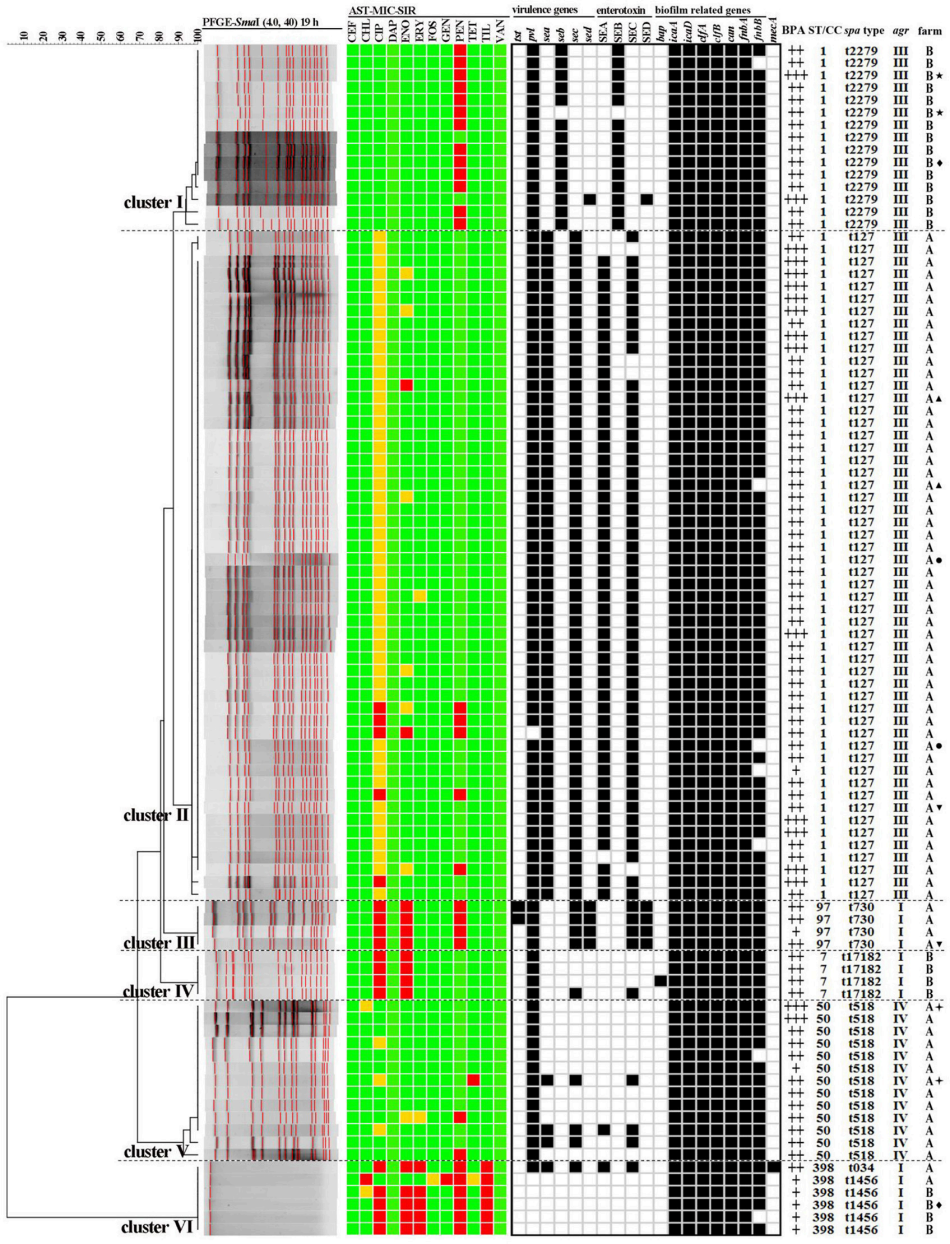
Dendrogram of PFGE patterns and antimicrobial susceptibility testing (AST), virulence genes, enterotoxin production, biofilm and adhesion related genes, *mecA* gene, and molecular characterization of 96 *S. aureus* isolates cultured from raw milk in Beijing China. Ninety-six isolates were grouped into 6 clusters (cluster I-VI) by PFGE patterns and all had more than 92% similarity. The results of AST were showed in different colors according to the MIC values of isolates to different antimicrobial agents. Green squares indicate susceptibility; yellow squares indicate intermediate; and red squares indicate resistance. The detection of virulence genes, enterotoxin production, biofilm and adhesion related genes, and *mecA* gene were summarized by a heat map. Black squares denote that the studied genes were detected in those isolates, or those isolates could produce those types of enterotoxins. White squares denote that those isolates lack these studied genes or could not produce those types of enterotoxins. BPA represents biofilm production ability. ST/CC represents sequence type of MLST and the clone complex (CC) of this ST. *agr* represents *agr* types. Antimicrobial compounds used are abbreviated as follows: TIO, Ceftiofur; CHL, chloramphenicol; CIP, ciprofloxacin; DAP, daptomycin; ENO, enrofloxacin; ERY, erythromycin; FOS, fosfomycin; GEN, gentamycin; PEN, penicillin; TET, tetracycline; TIM, tilmicosin; VAN, vancomycin. The same symbols beside farm number of •, ▴, ✦, ▾, ⋆, and ♦ represent isolates cultured from M11, M17, M23, M34, M87, and M91, respectively.

Of the 195 raw milk samples, 90 (46.2%, 90/195) were confirmed with *S. aureus*, and in all 96 isolates were obtained in this study (Table 2). Twelve isolates cultured from six samples (2 isolates were cultured per samples), respectively, were included in this study, as both strains of each sample were subsequently found to have different genetic patterns and/or phenotypes (Table 3 and Figure 1). Of the 90 *S. aureus*-positive samples, 71 of 147 (48.2%) and 19 of 48 (39.6%) raw milk samples collected from farm A and farm B respectively, were positive for *S. aureus*. Meanwhile, 73 and 23 *S. aureus* isolates were obtained from samples collected from farm A and farm B, respectively. Additionally, one *S. aureus* isolate (1%, 1/96) cultured from farm A was then identified to harbor the *mecA* gene, thereby classifying it as a MRSA isolate (Table 2 and Figure 1).

**Table 2:** Prevalence of *S. aureus* in raw milk in Beijing.

**Farm**	**No. of samples**	**No. (%) of samples with confirmed *S. aureus***	**No. of *S. aureus* isolates**	**No. (%) of MRSA isolates**
A	147	71 (48.2%)	73	1 (1.4%)
B	48	19 (39.6%)	23	ND^*^
Total	195	90 (46.2%)	96	1 (1%)

* *ND means no detection*.

**Table 3:** Characteristics of isolates cultured from the same samples^a^.

**Sample ID**	**Isolates**	**Genotype patterns**	**Virulence genes**	**Biofilm related genes**	**Antimicrobial resistance**	**Enterotoxin production**	**Biofilm formation**	**Farms**
M11	M11-1	PFGE cluster II-CC1-ST1-t127-*agr* III	*sea-sec*	*icaA*-*icaD*-*clfA-clfB*-*can*-*fnbA-fnbB*	-	SEA-SEC	++	A
	M11-2	PFGE cluster II-CC1-ST1-t127-*agr* III	*pvl-sea-sec*	*icaA*-*icaD*-*clfA-clfB*-*can*-*fnbA-fnbB*	-	SEA-SEC	++	A
M17	M17-1	PFGE cluster II-CC1-ST1-t127-*agr* III	*pvl-sea-sec*	*icaA*-*icaD*-*clfA-clfB*-*can*-*fnbA-fnbB*	-	SEA-SEC	+++	A
	M17-2	PFGE cluster II-CC1-ST1-t127-*agr* III	*pvl-sea-sec*	*icaA*-*icaD*-*clfA-clfB*-*can*-*fnbA*	-	SEA-SEC	++	A
M23	M23-1	PFGE cluster V-CC50-ST50-t518-*agr* IV	*pvl*	*icaA*-*icaD*-*clfA-clfB*-*can*-*fnbA-fnbB*	-	-	+++	A
	M23-2	PFGE cluster V-CC50-ST50-t518-*agr* IV	*pvl-sea-sec*	*icaA*-*icaD*-*clfA-clfB*-*can*-*fnbA-fnbB*	TET	SEC	++	A
M34	M34-1	PFGE cluster II-CC1-ST1-t127-*agr* III	*pvl-sea-sec*	*icaA*-*icaD*-*clfA-clfB*-*can*-*fnbA-fnbB*	-	SEA-SEC	++	A
	M34-2	PFGE cluster III-CC97-ST97-t730-*agr* I	*pvl-sec-sed*	*icaA*-*icaD*-*clfA-clfB*-*can*-*fnbA-fnbB*	PEN-CIP-ENO	SEC-SED	++	A
M87	M87-1	PFGE cluster I-CC1-ST1-t2279-*agr* III	*pvl*-*seb*	*icaA*-*icaD*-*clfA-clfB*-*can*-*fnbA-fnbB*	PEN	SEB	+++	B
	M87-2	PFGE cluster I-CC1-ST1-t2279-*agr* III	*pvl*	*icaA*-*icaD*-*clfA-clfB*-*can*-*fnbA-fnbB*	PEN	-	++	B
M91	M91-1	PFGE cluster I-CC1-ST1-t2279-*agr* III	*pvl*-*seb*	*icaA*-*icaD*-*clfA-clfB*-*can*-*fnbA-fnbB*	PEN	SEB	++	B
	M91-2	PFGE cluster VI-CC398-ST398-t14156-*agr* I	-	*icaA*-*icaD*-*clfA-clfB*-*can*-*fnbA-fnbB*	PEN-CIP-ENO-ERY-TIL	-	+	B

a *“-”means that isolates did not have this genotype or phenotype*.

### Antimicrobial susceptibility

Table 4 shows the antimicrobial susceptibility results for the tested isolates. Of the 96 *S. aureus* isolates tested, resistance was most frequently observed to penicillin (31.3%, 30/96), followed by ciprofloxacin (18.8%, 18/96) and enrofloxacin (15.6%, 15/96), and to a lesser extent tilmicosin (6.3%, 6/96), erythromycin (5.2%, 5/96), gentamycin (1.0%, 1/96), chloramphenicol (1.0%, 1/96), and tetracycline (1.0%, 1/96). Isolates from farm B showed significantly higher resistance to penicillin (73.9%), ciprofloxacin (34.8%), enrofloxacin (34.8%), tilmicosin (17.4%), and erythromycin (17.4%) than those from farm A (*p* < 0.05; Table 4). All *S. aureus* isolates were susceptible to ceftiofur, daptomycin, and vancomycin. Notably, 52 (54.2%, 52/96) and seven (7.3%, 7/96) isolates, all of which were cultured from farm A, expressed an intermediate phenotype to ciprofloxacin and enrofloxacin, respectively. Meanwhile, for the top three resistant phenotypes to penicillin, ciprofloxacin, enrofloxacin, the MIC_50_ and MIC_90_ were measured at 0.06- and 8-μg/mL, 2- and 8-μg/mL, 0.5 and 4-μg/mL, respectively. Additionally, thirty-seven isolates (38.5%, 37/96) showed resistant to at least one antimicrobial and 6 isolates (6.3%, 6/96) showed resistant to ≥3 classes (MDR) (Tables 4, 5 and Figure 1). Totally, nine resistance patterns were identified, wherein PEN (16.7%, 16/96), PEN-CIP-ENO-ERY-TIL (5.2%, 5/96) and PEN-CIP-ENO (5.2%, 5/96) were the top three frequently identified patterns. Greater diversity among the resistance patterns from farm A (8 patterns) than those from farm B (3 patterns), were noted (Table 5 and Figure 1). PEN-CIP-ENO-ERY-TIL, and PEN were more frequently detected from farm B than from farm A (*p* < 0.05), while PEN-CHL-GEN-TIL, PEN-CIP-ENO, PEN-CIP, CIP, ENO, and TET were only identified in farm A and CIP-ENO only in farm B.

**Table 4:** Antimicrobial susceptibility of the study isolates to eight of the 12 antimicrobial agents tested.

**Antimicrobials**	**MIC_50_**	**MIC_90_**	**Range**	**Resistant, no. of isolates (%)**			**Intermediate, no. of isolates (%)**			**Susceptible, no. of isolates (%)**		
				**Farm A**	**Farm B**	**Total**	**Farm A**	**Farm B**	**Total**	**Farm A**	**Farm B**	**Total**
Penicillin	0.06	8	0.06—32	13(17.8)	17(73.9)^*^	30(31.3)	0(0)	0(0)	0(0)	60(82.2)	6(26.1)	66(68.8)
Ciprofloxacin	2	8	0.125—16	10(13.7)	8(34.8)^*^	18(18.8)	52(71.2)	0(0)	52(54.2)	11(15.1)	15(65.2)	26(27.1)
Enrofloxacin	0.5	4	0.125—32	7(9.6)	8(34.8)^*^	15(15.6)	7(9.6)	0(0)	7(7.3)	59(80.1)	15(65.2)	74(77.1)
Tilmicosin	2	2	0.5—64	2(2.7)	4(17.4)^*^	6(6.3)	0(0)	0(0)	0(0)	71(97.3)	19(82.6)	90(93.8)
Erythromycin	0.25	0.25	0.125—16	1(1.4)	4(17.4)^*^	5(5.2)	2(2.7)	0(0)	2(2.1)	70(95.9)	19(82.6)	89(92.7)
Gentamycin	1	1	0.5—64	1(1.4)	0(0)	1(1)	0(0)	0(0)	0(0)	72(98.6)	23(100)	95(99)
Chloramphenicol	8	8	0.5—128	1(1.4)	0(0)	1(1)	1(1.4)	1(4.3)	2(2.1)	71(97.3)	22(95.7)	93(96.9)
Tetracycline	0.5	0.5	0.25—64	1(1.4)	0(0)	1(1)	1(1.4)	0(0)	1(1)	71(97.3)	23(100)	94(97.9)
Fosfomycin	32	64	0.5—256	0(0)	0(0)	0(0)	1(1.4)	0(0)	1(1)	72(98.6)	23(100)	95(99)
Ceftiofur	0.5	2	0.25—64	0(0)	0(0)	0(0)	0(0)	0(0)	0(0)	73(100)	23(100)	96(100)
Daptomycin	0.5	1	0.06—16	0(0)	0(0)	0(0)	0(0)	0(0)	0(0)	73(100)	23(100)	96(100)
Vancomycin	0.5	1	0.06—128	0(0)	0(0)	0(0)	0(0)	0(0)	0(0)	73(100)	23(100)	96(100)

* *p < 0.05*.

**Table 5:** Phenotypes and genotypes of 96 *S. aureus* isolates tested in this study.

**Phenotypes or genotypes tested in this study**		**No. of isolates (%)**		
		**Farm A**	**Farm B**	**Total**
Antimicrobial resistance patterns	PEN	3(4.1)	13(56.5)^*^	16(16.7)
	CIP	1(1.4)	0(0)	1(1)
	ENO	1(1.4)	0(0)	1(1)
	TET	1(1.4)	0(0)	1(1)
	PEN-CIP	3(4.1)	0(0)	3(3.1)
	CIP-ENO	0(0)	4(17.4)	4(4.2)
	PEN-CIP-ENO	5(6.8)	0(0)	5(5.2)
	PEN-CHL-GEN-TIL	1(1.4)	0(0)	1(1)
	PEN-CIP-ENO-ERY-TIL	1(1.4)	4(17.4)^*^	5(5.2)
	ND	57(78.1)	2(8.7)	59(61.5)
Virulence genes	*tst*	2(2.7)	0(0)	2(2.1)
	*pvl*	71(97.3)^*^	19(82.6)	90(93.8)
	*sea*	58(79.5)	0(0)	58(60.4)
	*seb*	0(0)	14(60.9)	14(14.6)
	*sec*	62(84.9)^*^	1(4.3)	63(65.6)
	*sed*	4(5.5)	1(4.3)	5(5.2)
	*see*	0(0)	0(0)	0(0)
	ND	1(1.4)	4(17.4)	5(5.2)
Virulence gene patterns	*pvl*	10(13.7)	4(17.4)	14(14.6)
	*pvl*-*seb*	0(0)	13(56.5)	13(13.5)
	*pvl*-*sec*	0(0)	1(4.3)	1(1)
	*sea*-*sec*	1(1.4)	0(0)	1(1)
	*pvl*-*sea*-*sec*	57(78.1)	0(0)	57(59.4)
	*pvl*-*seb*-*sed*	0(0)	1(4.3)	1(1)
	*pvl*-*sec*-*sed*	2(2.7)	0(0)	2(2.1)
	*tst*-*pvl*-*sec*-*sed*	2(2.7)	0(0)	2(2.1)
	ND	1(1.4)	4(17.4)	5(5.2)
Enterotoxin production	SEA	53(72.6)	0(0)	53(55.2)
	SEB	0(0)	14(60.9)	14(14.6)
	SEC	58(79.5)^*^	1(4.3)	59(61.5)
	SED	4(5.5)	1(4.3)	5(5.2)
	SEE	0(0)	0(0)	0(0)
	ND	11(15.1)	8(34.8)	19(19.8)
Enterotoxin production patterns	SEA	4(5.5)	0(0)	4(4.2)
	SEB	0(0)	13(56.5)	13(13.5)
	SEC	5(6.8)	1(4.3)	6(6.3)
	SEA-SEC	49(67.1)	0(0)	49(51)
	SEB-SED	0(0)	1(4.3)	1(1)
	SEC-SED	4(5.5)	0(0)	4(4.2)
	ND	11(15.1)	8(34.8)	19(19.8)
Biofilm related genes	*icaA*-*icaD*-*clfA-clfB*-*can*-*fnbA*	5(6.8)	2(8.6)	7(7.3)
	*icaA*-*icaD*-*clfA-clfB*-*can*-*fnbA-fnbB*	68(93.2)	20(86.9)	88(91.7)
	*bap*-*icaA*-*icaD*-*clfA-clfB*-*can*-*fnbA-fnbB*	0(0)	1(4.3)	1(1)
Biofilm production ability^a^	+ (range of OD: 0.913-1.196)	4(5.5)	4(17.39)	8(8.3)
	++ (range of OD: 1.246-1.797)	53(72.6)	17(73.9)	70(72.9)
	+++ (range of OD: 1.807-2.156)	16(21.9)	2(8.7)	18(18.8)
*agr* types	I	6(8.2)	8(34.8)	14(14.6)
	II	0(0)	0(0)	0(0)
	III	54(74)	15(65.2)	69(71.9)
	IV	13(17.8)	0(0)	13(13.5)
MLST	CC1-ST1	54(74)	15(65.2)	69(71.9)
	CC7-ST7	0(0)	4(17.4)	4(4.2)
	CC50-ST50	13(17.8)	0(0)	13(13.5)
	CC97-ST97	4(5.5)	0(0)	4(4.2)
	CC398-ST398	2(2.7)	4(17.4)	6(6.3)
*spa* typing	t034	1(1.4)	0(0)	1(1)
	t127	54(74)	0(0)	54(56.3)
	t518	13(17.8)	0(0)	13(13.5)
	t730	4(5.5)	0(0)	4(4.2)
	t2279	0(0)	15(65.2)	15(15.6)
	t14156	1(1.4)	4(17.4)	5(5.2)
	t17182	0(0)	4(17.4)	4(4.2)
Genotype patterns	PFGE cluster I-CC1-ST1-t2279-*agr* III	0(0)	15(65.2)	15(15.6)
	PFGE cluster II-CC1-ST1-t127-*agr* III	54(74)	0(0)	54(56.3)
	PFGE cluster III-CC97-ST97-t730-*agr* I	4(5.5)	0(0)	4(4.2)
	PFGE cluster IV-CC7-ST7-t17182-*agr* I	0(0)	4(17.4)	4(4.2)
	PFGE cluster V-CC50-ST50-t518-*agr* IV	13(17.8)	0(0)	13(13.5)
	PFGE cluster VI-CC398-ST398-t034-*agr* I	1(1.4)	0(0)	1(1)
	PFGE cluster VI-CC398-ST398-t14156-*agr* I	1(1.4)	4(17.4)	5(5.2)

a *Quantification of biofilm formation by optical density (OD) determination: (+++): strong biofilm producers (OD570 > 1.8), (++): moderate biofilm producers (1.8 > OD570 > 1.2), (+): weak biofilm producers (1.2 > OD570 > 0.6)*;

* *p < 0.05*.

### Presence of virulence and biofilm related genes

Of the 96 *S. aureus* isolates tested, 91 (94.8%) were detected to have one or more virulence genes, and 6 virulence genes (*tst, pvl, sea* to *sed*) were identified with no *see* genes amplified, by PCR in this study (Table 5 and Figure 1). The 4 SEs genes were detected in 80.2% (77/96) of all 96 isolates. The three most frequently detected virulence genes were *pvl* (93.8%, 70/96), *sec* (65.6%, 63/96), and *sea* (60.4%, 58/96), followed by *seb* (14.6%, 14/96), *sed* (5.2%, 6/96), and *tst* (2.1%, 2/96). Prevalence rates of the *pvl* and *sec* genes from farm A (97.3% and 84.9% respectively) were higher than those from farm B (82.6 and 4.3% respectively) (*p* < 0.05). While, the *ts*t and *sea* genes were only identified in farm A, and the *seb* gene was only identified in farm B (Table 5). In total, eight different virulence gene patterns were observed. Among all patterns, the *pvl*-*sea*-*sec* (59.4%, 57/96) was common, followed by *pvl* (14.6%, 14/96), *pvl*-*seb* (13.5%, 13/96). The *pvl*-*sec*-*sed* and *tst*-*pvl*-*sec*-*sed* patterns were found in 2.1% (2/96 each) of all 96 isolates, respectively, while *pvl-sec, sea-sec*, and *pvl-seb-sed* were found in 1% (1/96 each) of all 96 isolates, respectively (Table 5).

Table 5 lists the biofilm and adhesion related genes of the 96 *S. aureus* isolates recovered from farm A and farm B. The results show that the *icaA, icaD, clfA, clfB, can*, and *fnbA* genes were detected in all of the 96 isolates, while 7 isolates (5 from farm A and 2 from farm B) did not carry the *fnbB* gene and the *bap* gene was only detected in one isolate from farm B.

### Determination of enterotoxin production, and biofilm production ability

In total, 77 isolates were detected by PCR to have enterotoxin genes, while 53 (55.2%, 53/96), 14 (14.6%, 14/96), 59(61.5%, 59/96), and 5 (5.2%, 5/96) could produce SEA, SEB, SEC, and SED, respectively (Table 5). More than 90% of the SEs genes harboring *S. aureus* isolates could produce enterotoxins. Additionally, 54 (70.1%, 54/77) isolates simultaneously produced two types of enterotoxins (Table 5 and Figure 1). Moreover, the MRSA isolates harboring *sea* and *sec* genes also have the ability to producing both enterotoxins, SEA and SEC.

The microtiter plate assay showed that all 96 *S. aureus* from the two farms could produce biofilm, although at different intensities (Table 5 and Figure 1). Eight isolates (8.3%, 8/96), including 4 from farm A and farm B, were able to produce biofilm weakly; 68 strains (70.8%, 68/96), including 53 isolates from farm A and 17 isolates from farm B respectively, showed moderate biofilm formation; 18 strains (18.8%, 18/96), including 16 isolates from farm A and 2 isolates from farm B respectively, showed strong biofilm formation.

### MLST

**Figure 2:**
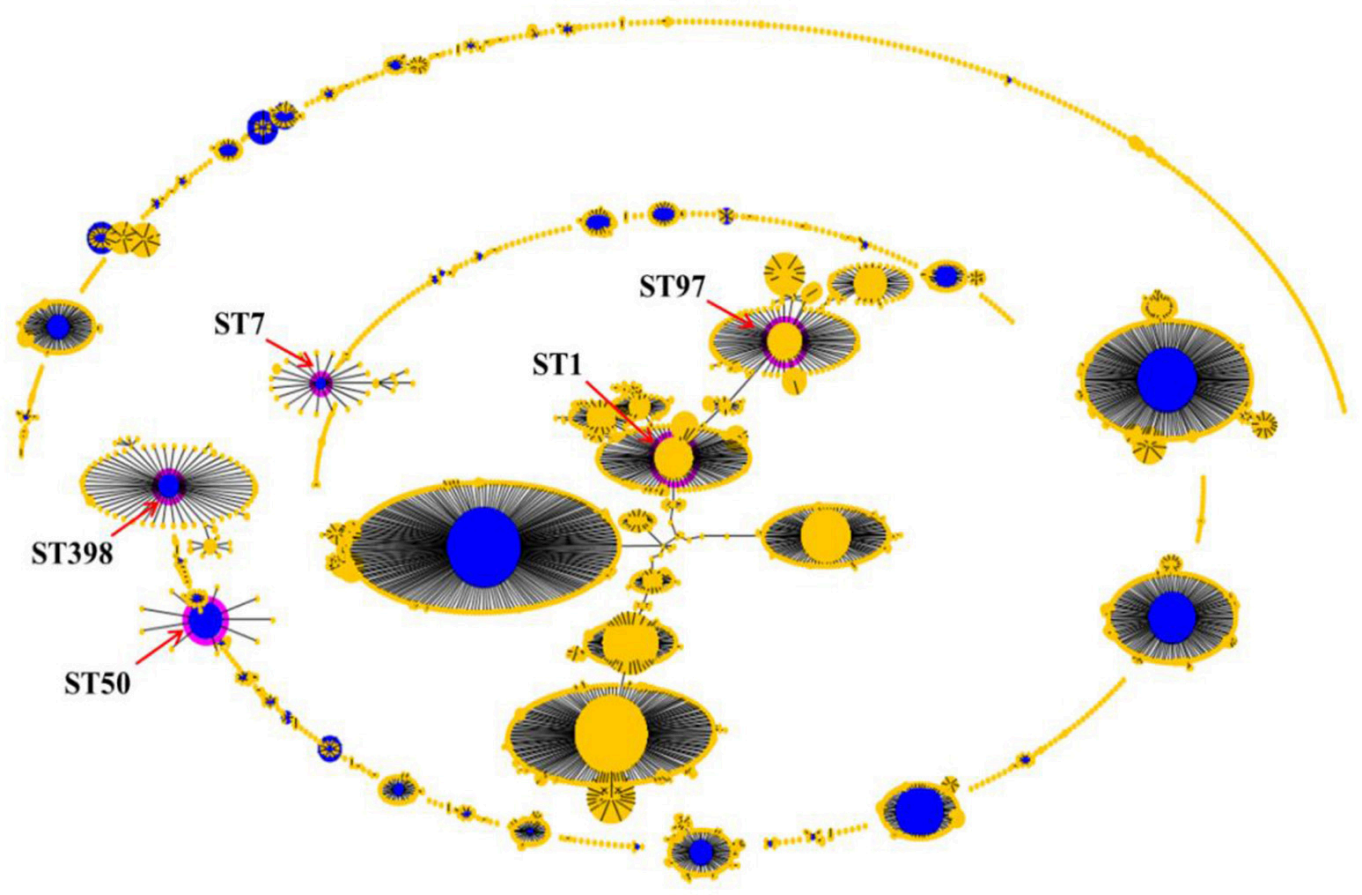
Population snapshot analyses by eBURST on 5779 *S. aureus* strains that belonged to 2766 STs in *S. aureus* MLST database. Pink circles labeled with red arrows and the ST names in black font are used for sequence types identified in this study.

**Figure 3:**
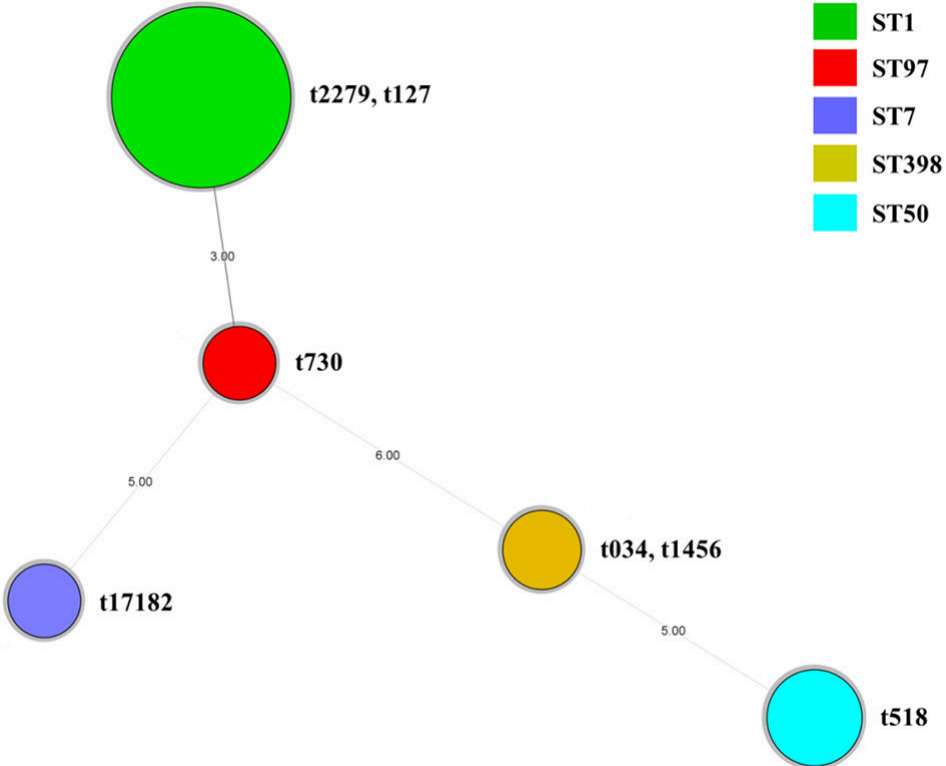
Minimum Spanning Tree of 96 *S. aureus* isolates based on the MLST data. Colors indicate ST of each node. The spa types of 96 *S. aureus* isolates were also showed within each node. Novel spa type identified in this study was t17182.

All 96 isolates were typed by MLST as shown in Table 5 and Figures 1–3. A total of 5 sequence types (STs) were identified (ST1, ST7, ST50, ST97, and ST398), which were further grouped into 5 CCs. In this study, CC1 was represented by ST1 (CC1-ST1) alone, being found as the most predominate sequence type (71.9%, 69/96) in both two farms, followed by CC50-ST50 (13.5%, 13/96), CC398-ST398 (6.3%, 6/96), and CC7-ST7 and CC398-ST398 (4.2%, 4/96 each). The clonal lineages of *S. aureus* isolates were further analyzed based on the sampling farms. As shown in Table 5 and Figure 1, four clonal lineages were identified from farm A, including CC1-ST1, CC50-ST50, CC97-ST97, and CC398-ST398. In contrast, three clonal lineages were identified from farm B, including CC1-ST1, CC7-ST7, and CC398-ST398.

### *spa* typing

A total of 7 *spa* types were obtained in all 96 *S. aureus*, with 1 novel *spa* type (t17182) identified (Table 5 and Figure 1). The most prevalent *spa* type was t127 (56.3%, 54/96) and this was associated with isolates cultured from farm A. In addition to t127, four other *spa* types were also found in isolates from farm A (t518, t730, t034, and t14156). Meanwhile, Isolates from farm B were defined by 3 *spa* types, including t2279, t14156, and t17182. Based on MLST, isolates of the sequence types ST7, ST50, and ST97 had their own identical *spa* types (ST50-t518, ST97-t730, and ST7-t17182) (Table 5 and Figures 1, 3). However, there were some exceptions that several isolates owned the identical sequence type but different *spa* types (ST1-t127/t2279, ST398-t034/t1456) (Table 5 and Figures 1, 3).

### *agr* genotyping

The distribution of *agr* alleles among the 96 isolates is provided in Table 5. Using a multiplex-based PCR, *agr* alleles were successfully identified in 96 isolates. The *agr* III genotype was predominant, representing 71.9% (69/96) of the isolates and was the prevailing *agr* type regardless of the sampling farms of *S. aureus* isolates, followed by *agr* I (14.6%, 14/96) and *agr* VI (13.5%, 13/96). No *agr* II type was detected among all 96 isolates. Furthermore, all 14 isolates with *agr* I were discriminated into three STs and four *spa* types (ST7-t17182, ST97-t730, ST398-t034, and ST398-t1456). All 69 isolates with *agr* III with the same sequence type were discriminated into two *spa* types (ST1-t2279 and ST1-t127). However, all 13 isolates with *agr* IV had the identical sequence type and *spa* type (ST50-t518) (Table 5 and Figure 1).

### PFGE sub-typing and identification of major clones

Among 96 isolates subtyped by PFGE, six isolates (belonging to ST398) could not be typed by this method (Table 5 and Figure 1). The other 90 isolates were distinguished into 14 pulso types and then gathered into five PFGE clusters (Cluster I–V) based on more than 92% genetic similarity. The predominant PFGE cluster was cluster II and included 54 isolates all cultured from farm A, and which were differentiated into 4 pulso types. Fifty of these 54 isolates were found to sharing the same PFGE banding patterns. All isolates in cluster II were characterized as PFGE cluster II-CC1-ST1-t127-*agr* III. Cluster I included 15 isolates with 5 pulso types and included PFGE Cluster I-CC1-ST1-t2279-*agr* III. All 15 isolates in cluster-I were cultured from farm B. Four isolates from farm A were characterized as PFGE Cluster III-CC97-ST97-t730-*agr* I, while another 4 isolates from farm A were included in PFGE Cluster III-CC97-ST97-t730-*agr* I characterized as PFGE Cluster IV-CC7-ST7-t17182-*agr* I. Cluster V included 13 isolates with 3 pulso types that were designated as Cluster V-CC50-ST50-t518-*agr* IV. Moreover, 6 ST398 isolates that could not be digested with *Sma*I, were grouped as PFGE cluster VI in this study (Cluster VI-CC398-ST398-t034/t1456-*agr* I). The DI values of PFGE, *spa* typing, MLST, and *agr* typing of all 96 isolates were 0.701, 0.641, 0.463, and 0.448, respectively.

### Relationship between phenotypes and genotypes

The relationship between antimicrobial resistance, virulence, biofilm and molecular subtypes is shown in Figure 1. Each clonal complex had specific antimicrobial resistance, virulence, and biofilm characteristics. Isolates identified as CC1-ST1 clones and contained within PFGE cluster I-t2279-*agr* III were found to be resistance only to PEN with two isolates susceptible to all tested antimicrobial agents tested, followed by three virulence gene patterns denoted as as*pvl*-*seb*(13/15), *pvl*(1/15), and *pvl*-*seb*-*sed* (1/15). Isolates within PFGE cluster II-CC1-ST1-t127-*agr* III exhibited more resistant diversity including PEN-CIP (3/54), PEN-CIP-ENO (1/54), PEN (1/54), CIP (1/54), ENO (1/54), followed by two virulence gene patterns denoted as *pvl*-*sea*-*sec* (53/54) and *sea*-*sec* (1/54). All isolates in this cluster were un-susceptible to CIP. All isolates within PFGE cluster III-CC97-ST97-t730-*agr* I expressed resistance to PEN, CIP, and ENO, followed by two virulence gene patterns, *tst*-*pvl*-*sec*-*sed*(2/4) and *pvl*-*sec*-*sed*(2/4). The isolates identified as PFGE cluster IV-CC7-ST7-t17182-*agr* I showed resistant to CIP and ENO, followed by two virulence gene patterns, *pvl*(3/4) and *pvl*-*sec* (1/4). Only three isolates (3/13) with PFGE cluster V-CC50-ST50-t518-*agr* IV exhibited a resistance phenotype (2 resistant to PEN and 1 resistance to TET) and all 13 isolates in this cluster harbored the *pvl* gene, with three isolates also carrying the *sea* and *sec* genes. In contrast, isolates identified as CC398-ST398 expressed the greatest MDR in this study (5 patterns of PEN-CIP-ENO-ERY-TIL and 1 patterns of PEN-CHL-GEN-TIL). Moreover, the only MRSA isolate with CC398-ST398-t034-*agr* I harbored three virulence genes of *pvl, sea* and *sec*, whereas another 5 CC398-ST398 isolates identified as t1456-*agr* I were found to carry none of the tested virulence genes. Biofilm formation assay showed that this CC398-ST398-t1456-*agr* I clone was only able to produce biofilm weakly in this study.

## Discussion

*S. aureus* has been considered as an important cause of zoonotic disease and the potential transmission of MRSA between livestock and humans through close contact, handling and/or consumption of *S. aureus* infected food of animal origin (Kateete et al., 2013; Song et al., 2015; Pereyra et al., 2016). The infection of dairy herds and contamination of raw milk by *S. aureus*, especially those expressing a MDR phenotype and possessing the ability for produce biofilm and toxins including enterotoxin, TSST-1 and PVL, remains an important public health issue (Cavicchioli et al., 2015; Wang et al., 2016). The public health significance caused by this bacterium is manifested by food-borne poisoning outbreaks caused by dairy products contaminated by *S. aureus*, including one of the largest food-borne outbreaks on record involving 13,420 infected individuals in Japan (Asao et al., 2003; Hennekinne et al., 2012). Of note, food-borne infections attributed to *S. aureus* contaminated dairy foods are also frequently reported in China (Rong et al., 2017). Additionally, the economic cost burden to the dairy farms is considerable; mastitis in dairy cow can result in reductions in milk yield, treatment expense and/or culling in sometimes (Hennekinne et al., 2012). This study investigated the prevalence, genetic diversity, antimicrobial resistance phenotypes, carriage of staphylococcal virulence factors along with testing the capacity of these isolates to produce biofilm and the 5 classical enterotoxins (SEA to SEE). All of these *S. aureus* were isolated from raw milk samples taken in 2 dairy farms in Beijing, China. Acquisition of the prevalence and characteristics of *S. aureus* isolated from raw milk would be helpful to obtain the antimicrobial resistance and virulence markers as well as predominant clones which can help prevent and control the *S. aureus* contamination in dairy herd and protect the end consumer.

In the present study, 46.2% (90/195) of raw milk samples taken from dairy cows with mastitis were positive for *S. aureus*. This prevalence is similar to a recent report in China and other reports in Brazil and Italy (Cavicchioli et al., 2015; Li et al., 2015; Giacinti et al., 2017). However, another recent study reported that the prevalence of *S. aureus* in raw milk of health cows in Beijing was 22.0% (Liu et al., 2017). Overall, our data indicate that *S. aureus* is common and frequently detected in the raw milk of dairy cows with mastitis in Beijing, China. Further research is needed to explore methods of controlling *S. aureus* occurrence in raw milk.

In recent years, the emergence of MDR *S. aureus*, particularly MRSA, leading to animal and human infections, has become a growing public health concern (Li et al., 2015). In the current study, few resistances were detected among all 96 *S. aureus* (38.5% resistant to at least one antimicrobial), which were similar to those in Italy (39.4%) and Poland (23%), but much lower than two previous reports in Chinese (87% and 72.2%, respectively) and those in India (95%) (Li et al., 2015; Rola et al., 2015; Mistry et al., 2016; Giacinti et al., 2017; Liu et al., 2017). Moreover, only 6 isolates (6.3%) showed MDR that was lower than reports in other regions in China (Li et al., 2015; Liu et al., 2017). According to previous studies, penicillin-resistant *S. aureus* are the most prevalence isolates among raw milk and ranged from less than 10% to over 80% (Li et al., 2015; Rola et al., 2015; Liu et al., 2017). In this study, 31.3% of *S. aureus* were resistant to this antimicrobial agent. It was notable that ciprofloxacin- and enrofloxacin-resistant *S. aureus* were found to be the next most frequently detected resistance types in addition to penicillin. Both are fluoroquinolones, wherein ciprofloxacin a third generation fluoroquinolone is used at clinical level while enrofloxacin is specially used for veterinary applications in China (Hoang et al., 2017; Li J. et al., 2017). Once human and/or animals become infected with these resistant isolates, treatment failure using these two antimicrobials, is inevitable. Additionally, 54.2 and 7.3% of the isolates from farm A expressed an intermediate phenotype to ciprofloxacin and enrofloxacin, respectively. Meanwhile, isolates from farm B exhibited significantly higher resistance to a panel of antimicrobial compounds including penicillin, ciprofloxacin, enrofloxacin, tilmicosin, and erythromycin when compared to those from farm A (*p* < 0.05). Moreover, the resistance patterns were different between two farms in that PEN-CIP-ENO-ERY-TIL and PEN were more frequently detected from farm B compared with farm A (*p* < 0.05). These results suggested that the isolates from both farms may have their own resistance characteristics and the resistance patterns from farm A were more diverse than those from farm B (*p* < 0.05). Furthermore, it has been reported that rational management and appropriate usage of antimicrobial compounds in food-producing livestock is very important to control and prevent the spread of drug-resistant isolates (Jessen et al., 2017). All isolates in this study exhibited low-level resistance to other antimicrobial agents tested and similarly the MIC_50_ and MIC_90_ values were relatively low, a situation that is much different to previous reports in China and other countries (Li et al., 2015; Mistry et al., 2016; Liu et al., 2017). The relatively low rate of resistance and MDR isolates observed in this study could be due to the extensive farming systems and the strict management of the use of antimicrobial agents by the company.

MRSA is considered as major cause of hospital-acquired and community-acquired infections (Gopal and Divya, 2017). Additionally, the contaminated animal and associated products have been supposed to be a potential source of community-acquired MRSA (Gopal and Divya, 2017). Recently, the isolation of MRSA from raw milk and dairy products has been reported worldwide (Rola et al., 2016; Tarekgne et al., 2016; Basanisi et al., 2017). In this study, one *S. aureus* isolate (1.4%, 1/96) was identified as MRSA being confirmed by amplifying the *mecA* gene. The current study's prevalence reported for MRSA is lower than those reported previously in China or India (4.8–48.7%) (Li et al., 2015; Mistry et al., 2016; Liu et al., 2017). However, the potential MRSA transmission risk via the food chain, particularly by insufficient pasteurization milk, cannot be ignored.

With regard to the risk of pathogenicity, the presence of virulence genes among all 96 isolates was also assessed in this study. The classic enterotoxin SE determinants, of *S. aureus* are known to cause sporadic food-poisoning incidents or even food-borne outbreaks. It is reported that 89.7% isolates from cow milk related to mastitis carried one or more SEs genes (Song et al., 2015). In the current study, 80.2% of the isolates were positive for SE encoding genes and the *sec* (65.6%) and *sea* (60.4%) genes were the most frequently detected. This finding is similar to those in previous reports from China and Australia, whereas the *sed* gene was mainly detected among isolates from raw milk samples in Poland (Rola et al., 2015; Song et al., 2015; McMillan et al., 2016; Liu et al., 2017). Meanwhile, another Chinese study reported that the *seb* gene was the most commonly detected (Cheng et al., 2016). Additionally, the prevalence rates of the *sec* gene from farm A (84.9%) was higher than from farm B (4.3%) (*p* < 0.05). While, the *sea* gene was only found in farm A, and the *seb* gene was only found in farm B. Therefore, the different prevalence rates observed among all SE genes could be due to the fact that these isolates originated in geographically diverse locations. According to previous reports, the *see* gene was rarely present in raw milk or even retail food in China, and similarly, this marker was not detected in this study. Notably, the *pvl*-encoding gene showed a very high prevalence (93.8%) in the tested isolates, which was similar to previous reports (Esposito et al., 2013; Aires-de-Sousa, 2017). It was reported that the *pvl*-encoding gene were present at a high prevalence among methicillin-sensitive isolates and the Livestock-associated MRSA (LA-MRSA) isolates positive with PVL mostly originated from humans (Price et al., 2012; Wardyn et al., 2012). Two isolates in this study were identified to have the *tst* gene, which could cause severe clinical diseases (Xie et al., 2011). Our data highlight the necessity to identify virulence factors among pathogenic *S. aureus*.

Several studies examined for the presence of SEs genes among *S. aureus* cultured from raw milk and their food products (Asao et al., 2003; Song et al., 2015; Cheng et al., 2016; Liu et al., 2017). However, few reports assessed the enterotoxin producing capacity of these isolates in China. To our best knowledge, this study firstly reported the production of 4 classic SEs in raw dairy milk in China. The results showed that >90% of the SEs (*sea* to *sed*) genes carried *S. Aureus* isolates could produce enterotoxins. Additionally, 54 (70.1%, 54/77) of the SE gene carrying *S. aureus* simultaneously produced two types of enterotoxins, including one MRSA isolate (positive for SEA and SEC). Once enterotoxins were already produced, and these can generally retain their biological activity even after heat treatment (Cavicchioli et al., 2015). Thus, it is necessary to develop measures to eliminate the contamination of this bacterium in dairy products.

The study also investigated the distribution of biofilm and adhesion related genes among all isolate, some of which are also related to bacterial virulence (Rasmussen et al., 2013). In this study, all 96 isolates harbored the *icaAD, fnbA, clfAB*, and *cna* genes and 92.7% of the isolates harbored the *fnbB* gene. In contrast the *bap* gene was only detected in one isolate. Thus, these isolates have the ability to form biofilm a feature that suggests these bacteria have the potential to persist in this environment. The ability to form biofilms helps *S. aureus* to persist in infections and subclinical and clinical cases of bovine mastitis (Dhanawade et al., 2010). In the present study all 96 *S. aureus* isolates could form biofilms as determined by the microtiter plate assay described above, and these findings agree with a previous report from Argentina but being higher in number than reported in a similar study from Brazil (Lee et al., 2014; Pereyra et al., 2016). The high incidence of biofilm-producing *S. aureus* isolates in this study suggests the necessary for dairy farms to improve the quality assurance systems, in order to decrease and eliminate these isolates.

Our data also highlighted the diverse genetic backgrounds of the *S. aureus* from raw milk by MLST, *spa* typing, *agr* typing and PFGE sub-typing. Since the MLST genotyping for *S. aureus* was first reported, it has been widely used in epidemiological analysis of *S. aureus* infection and associated food poisoning outbreaks (Enright et al., 2000). In this study, five sequence types were obtained by MLST and each was further grouped into a clonal complexes. CC1-ST1 was the predominant clone (71.9%, 69/96), followed by CC50-ST50, CC398-ST398, CC7-ST7, and CC398-ST398, all of which have been reported in raw milk in China, previously (Song et al., 2015). Moreover, the ST1 and ST97 lineages were also detected frequently from bovine milk worldwide, while ST398, the most common livestock-associated MRSA type, has been already found in both food-producing animal and human species (Mistry et al., 2016; Gopal and Divya, 2017). Six isolates were identified as ST398 including the only one detected as a MRSA strain in this study. It was reported that MRSA ST398 is the most prevalent clone in Europe and North America, whereas methicillin-susceptible *S. aureus* (MSSA) ST398 was predominant in Asian regions (Asai et al., 2012; Yan et al., 2014). In total, six known *spa* types (t034, t127, t518, t730, t1456, and t2279) and 1 newly identified *spa* type (t17182) were identified in this study. A previous study also observed *spa* diversity among the STs although some *spa* types corresponded with either an ST or a CC (Chao et al., 2015). The *spa* types, t127 and t2279, have been reported as community-associated clones previously, and these were the top two frequently distributed genotypes among raw milk samples where all isolates of both types were identified as ST1 (Song et al., 2015). Considering the transmission of bacterial species between humans and livestock is increasingly being detected in farm workers in several countries (Huijsdens et al., 2006; Kateete et al., 2013), a recent study showed that the t127 clone could be present in cows, humans and environments (Papadopoulos et al., 2018). Although isolates of this *spa* type exhibited less antimicrobial resistance in this study, the potential of biofilm and enterotoxin producing would lead to persistent existence and subsequent contamination. Therefore, this clone could be important source of contaminations in cow farms, leading to quickly spread and large infections in both dairy herd and human community.

Isolates of ST398 types corresponded to one t034 (MRSA) and 5 to t1456 (MSSA) along with each of the other STs being linked to sole *spa* type. Of note, the ST398-t1456 MSSA was firstly identified in China, while the ST398-t1456 clone was related to LA-MRSA in Europe (Köck et al., 2013). Furthermore, the newly identified *spa* type t17182 corresponded to ST7, which has been reported to be related to bovine mastitis (Li T. et al., 2017). Moreover, ST50-t518 found in this study was reported to be mainly present in bovines in Denmark (Hasman et al., 2010). The other *spa* type t730, has been less frequently detected then before, and corresponded to the bovine milk-associated sequence type ST97 (Gopal and Divya, 2017). In this study *agr* type III was the most predominant *agr* type (71.9%) among *S. aureus* isolates, which is in accordance with a previous report from Brazil (48.2%) (Silva et al., 2013). However, *agr*I and *agr* II could be predominant types according to previous reports (Fabres-Klein et al., 2015; Khoramrooz et al., 2016; Mistry et al., 2016). Only 14.6 and 13.5% of our isolates were identified as *agr* I and IV respectively, which are lower than previous reports (Fabres-Klein et al., 2015; Mistry et al., 2016). Similar to other studies the *agr* II was not identified in the current study (Fabres-Klein et al., 2015; Khoramrooz et al., 2016; Mistry et al., 2016).

PFGE is generally recognized as the current gold standard method, and it has been widely used in genotyping of various bacteria including bovine mastitis associated *S. aureus* (De Oliveira et al., 2000; McMillan et al., 2016). Previous studies demonstrated that different clonal lineages may exhibit specific patterns of antimicrobial resistance and contain various virulence factors (Hata et al., 2010; Song et al., 2015). In this study, isolates of the PFGE cluster II (56.3%) and cluster I (15.6%) were the most frequently detected. All belonged to ST1 (CC1), t127/2279 along with the *agr* type or *agr* III which were grouped in these two clusters. The *agr* system is related to the regulation of virulence factors and different *agr* groups may have specific virulence patterns (Melchior et al., 2009; Khoramrooz et al., 2016). This study showed that isolates of *agr* III of represented by two clones (PFGE Cluster I/II-CC1-ST1-t127/2279), carried more virulence genes than those of *agr* I and *agr* IV types, suggesting that *agr* profiles may be associated with the virulence potential of *S. aureus*. Furthermore, isolates in PFGE Cluster II-CC1-ST1-t127-*agr* III exhibited the most diversities of antimicrobial resistant, while isolates in PFGE Cluster I-CC1-ST1-t2279-*agr* III was only resistant to PEN. Of note, the 5 MSSA-ST398-t1456-*agr* I isolates expressed the most MDR patterns but with no virulence genes and showed weakly biofilm formation, whereas the MRSA-ST398-t034-*agr* I clone expressed MDR and virulence (*pvl*-*sea*-*sec*) as well as showing moderate biofilm formation in this study. All isolates within PFGE cluster III-CC97-ST97-t730-*agr* I clone were resistant to PEN, CIP, and ENO, while all isolates in the PFGE cluster IV-CC7-ST7-t17182-*agr* I showed resistant to CIP and ENO. Geographically, isolates from farm A and farm B were well distinguished phylogenetically in this study. It is interesting that we found different isolates from the same mastitic milk sample that showed different genotypes or phenotypes in this study, which confirmed the fact that different clones could colonize in one host, making it harder to eliminate and control *S. aureus* infections in dairy cows.

## Conclusions

In summary, our research provides detailed epidemiological survey on the prevalence of *S. aureus* in raw milk of dairy cows with mastitis in Beijing, China. This study demonstrated a rather high prevalence of *S. aureus* with enterotoxigenic and biofilm forming abilities that may contribute to *S. aureus* persisting in the dairy farms leading to severe infections and subsequent food poisoning. To the best of our knowledge, this study firstly reported the classic SEs production in raw milk from cows in China. However the percentage of MDR and MRSA isolates was low in this study, their pathogenicity and transmission risk cannot be ignored. Of note, it is necessary to control and eliminate the present of MDR, enterotoxigenic and biofilm formatting *S. aureus* in raw milk. Additionally, our study also demonstrated the genetic diversity these isolates. Results of the present study highlight the dominant genetic lineages of livestock associated found not only in China but also worldwide. Although new *spa* type variants were found, their lineage related sequence type suggested that these strains may also associate with bovine mastitis. Significant differences genetic diversity along with antimicrobial resistance, virulence factors and biofilm formation were observed for *S. aureus* isolates from raw milk. It was shown that *S. aureus* with similar genetic characteristic displayed specific antimicrobial resistance patterns, virulence gene profiles, biofilm formations and geographic features and different clones could colonize in one dairy host. Therefore, monitoring the genotypes of *S. aureus* in dairy cow would give assistance to distinguish prevalent clones, which can help dairy farms develop control measures for mastitis caused by *S. aureus*.

## Availability of data and materials

The aggregate data supporting findings contained within this manuscript will be shared upon request submitted to the corresponding author.

## Author contributions

WW, ZB, XL, FL, and SF designed experiments. TJ, ZP, JX, and LY carried out experiments. WW and XL analyzed experimental data. WW, ZB, FL, and SF wrote the manuscript.

### Conflict of interest statement

The authors declare that the research was conducted in the absence of any commercial or financial relationships that could be construed as a potential conflict of interest.
